# Exploring the utilization of oat by-products in the circular bioeconomy: the potential for the development of extruded snacks enriched with β-glucan

**DOI:** 10.1186/s40643-026-01007-6

**Published:** 2026-01-27

**Authors:** Chen-Ru Lin, Ping-Hsiu Huang, Wen-Chang Chang

**Affiliations:** Department of Food Sciences, National Chiayi University, No.300 Syuefu Rd., Chiayi City, 600355 Taiwan

**Keywords:** Dietary fiber, Extrusion, Plant-based, Sustainability

## Abstract

The increasing prevalence of oat-based milk substitutes in the market has led to the generation of a substantial volume of β-glucan-enriched oat by-products (OBP) during production. However, the high moisture content of OBP poses challenges, as it is prone to decay and deterioration, making it difficult to recycle and reuse. This study aimed to pre-treat OBP using various drying methods and incorporate different ratios of brown rice with OBP in the extrusion process. The goal was to examine the effects of these variables on the physicochemical properties, textural characteristics, and digestibility of extruded snacks. Results showed that dried OBP was successfully incorporated into brown rice-based extruded snack formulations at levels of 5–15%. The radial expansion ratio (ER), bulk density (BD), and fracturability exhibited a negative correlation with the incorporation percentage, whereas hardness showed a positive correlation (*p* < 0.05). Additionally, the β-glucan and resistant starch (RS) contents of the extruded snacks increased significantly with higher OBP incorporation. The glycemic index (GI) of the extruded snacks decreased significantly, from 83.51 to 75.35, as the OBP content in the formulation increased. However, the sensory evaluation results showed that the consumer-based panelists agreed to incorporate 5% freeze-dried (FD) or complex drying (CD; autoclaving combined with hot-air drying) OBP into the extruded snacks. This study emphasizes the importance of carefully selecting incorporation ratios and employing suitable drying pre-treatments to achieve optimal sensory characteristics in extruded snacks.

## Introduction

Recently, there has been a notable surge in health consciousness and sustainable bioeconomy circle background, where the nutritional profile of extruded snacks has been enhanced by incorporating additional nutrients, bioactive elements, and by-products from food industries (grains’ bran, pomace, oilseed cake, and brewers spent must) sourced beyond cereals (Cheng et al. 2024a, b; Mohammed et al. 2024; Sumargo et al. 2016; Ying et al. 2017). Simultaneously, there is a pressing need to address the high levels of salt, fat, and sugar found in numerous snacks available in the market, with the aim of improving their overall nutritional profile (Grasso 2020). These advancements promote healthier dietary choices and serve as pivotal strategies in addressing the widespread prevalence of malnutrition and realizing zero hunger (Kalahal et al. 2024). Therefore, whether nutritional, economical, earth-friendly, or a combination of these, a specific and quantifiable added value should be provided to evaluate the practical contribution of by-products used rather than the repetitive substituting ingredients in similar recipes for pure publication work (Grasso 2020). However, the broader food business chain has been long and extensive, involving the agri-food industries (Auestad and Fulgoni 2015). The social responsibility approach of the established food industry is dedicated to providing products that are safe for consumption, consistent in quality, and offered at reasonable prices (Lucarini et al. 2020). However, this approach can also limit the industry’s capacity to allocate resources to research, development, and innovation compared to other sectors with greater profitability. Consequently, this scenario has given rise to the widespread view of the food industry as being traditional (Mansilla-Obando et al. 2024). Additionally, the reutilization of developed items and associated surplus resources facilitates a deeper understanding of the circular bioeconomy (Do et al. 2022). Notably, there are significant differences in the recycling of by-products between laboratory-scale and scaled-up circular production in terms of processed capacity, quantities, and environmental impact (C.-H. Lin et al. 2023a, b, c). It is imperative to refrain from embracing unrealistic one-size-fits-all approaches when utilizing these by-products and to consider their edibility and palatability (Cheng et al. 2023; Kruijssen et al. 2020; C.-H. Lin et al. 2023a, b, c). Therefore, apart from advocating for a healthy diet, these approaches can contribute progressively to promoting food security and sustainability towards achieving the United Nations’ Sustainable Development Goals (SDGs; 2: Zero hunger; 3: Good health and well-being; and 12: Responsible consumption and production) (Cooney et al. 2023; Y. D. Lin et al. 2023a, b, c).

Oat (*Avena sativa* L.) is one of the globally essential cereal crops, with an annual global production of about 25.13 million metric tonnes (Shahbandeh 2024), and it is extensively used in the food industry. Interestingly, although oat cultivation has remained stable in the past, market demand has soared in recent years due to the numerous health benefits of oats (Ma et al. 2021; Mahadevan et al. 2016). These benefits include lowering total cholesterol and low-density lipoprotein cholesterol (LDL), decreasing the risk of heart disease, and enhancing satiety, while both the yield and value of oat crops have experienced notable improvements (Ma et al. 2021; Mahadevan et al. 2016). Moreover, the escalating commercial availability of oats in beverages, healthy foods, cosmetics, and pharmaceuticals indicates a growing trend (Ma et al. 2021). Specifically, its macronutrients of 7–20% high-quality plant protein, dietary fiber (40% water-insoluble and 80% water-soluble), and high digestibility (90–94%), substantial amounts of β-glucan (4–8%) have been known to benefit from reducing the risks of all those chronic and metabolic diseases mentioned above (Ek et al. 2020; Jokinen et al. 2023; Yang et al. 2023). Moreover, incorporating this property into food recipes can be an effective supplement to compensate for the lack of fiber consumption in regular diets (Wu et al. 2024).

Recently, there has been a growing consumer trend toward plant-based protein beverages as a viable alternative to animal-based dairy products, with oat-based milk substitutes emerging as a prominent choice (Jokinen et al. 2021; McCarron et al. 2024; Silventoinen-Veijalainen et al. 2024). Notably, these plant-based products or animal-based substitutes have been recognized as sustainable for the environment, as these foods reduce negative environmental impacts, namely by contributing to the promotion of the aforementioned circular bioeconomy with the SDGs 2, 3, and 12 (Auestad and Fulgoni 2015; Mansilla-Obando et al. 2024). Specifically, the starch pasting and hydrolysis in oats affect the sensory quality of the end product, whereas the richness of dietary fiber and the insoluble particles derived from insoluble proteins cause a negative impact on the beverage’s properties (palatability and smoothness) (Sethi et al. 2016; Silventoinen-Veijalainen et al. 2024). It can also bring a sandy mouthfeel and cause sedimentation during the storage of the end products (McCarron et al. 2024; Sharma et al. 2024; Silventoinen-Veijalainen et al. 2024). However, in the commercially available manufacturing process, relatively larger particles are eliminated during beverage production to improve the beverage’s palatability, and the remaining liquid is then homogenized (McCarron et al. 2024; Sethi et al. 2016; Sharma et al. 2024). Regrettably, the necessary compromises, specifically the filtration of the OBP-enriched β-glucan, to meet consumer acceptability, have resulted in significant waste in terms of raw material costs and raised concerns for the circular bioeconomy. Therefore, considering the above issues, this study proposes to develop OBP into a palatable extruded snack using extrusion processing technology. By increasing the added value of OBP, we anticipate not only improving the digestive properties of starch but also transforming it into a functional snack.

## Materials and methods

### Materials

The brown rice (1st grade of Chinese National Standard 2424 (CNS 2019) was purchased from Golden Rice Castle Co., Ltd. (Chiayi, Taiwan). The OBP used in this study was provided by AGV Products Co. (Chiayi, Taiwan), specifically oats originating from Australia and harvested in 2022. Unless otherwise stated, all chemicals were purchased directly from Sigma-Aldrich^®^ (Merck KGaA, Darmstadt, Germany) and used without further processing.

### Extrusion processing parameters

The grain cake biscuit machine (Mibo-C1, YUAN CHUANG Food Machinery Co., Ltd., New Taipei City, Taiwan) was utilized in this study. The specific conditions were: mold hole diameter of 3 mm, screw speed of 50 HZ, feed rate at 16 rpm, sleeve temperature of 80 °C, and cutter speed of 2 rpm. The above conditions were obtained through preliminary experiments.

### Oat residues drying and extrusion treatment prior to processing

**Fig. 1:**
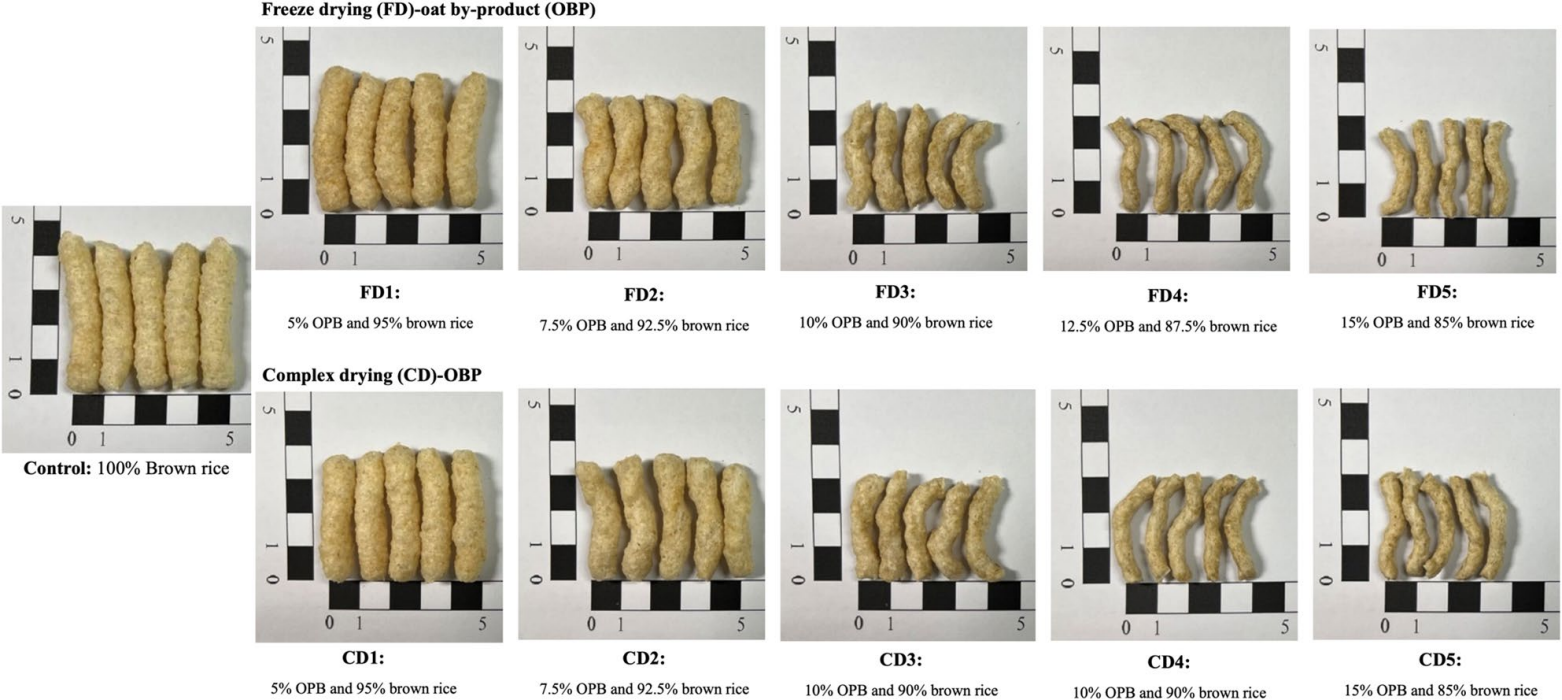
Effect of different ratios of freeze-dried (FD) and complex drying (CD; autoclaving combined with hot-air drying) of oat by-product (OBP) on the appearance of extruded snacks

The raw material OBP in this study was dried by freeze-drying (FD; at − 80 °C for 48 h) and complex drying [CD; autoclaving (121 °C, 15 min) combined with hot-air (50 °C) drying 24 h], respectively. Due to the high moisture content of OBP, both of which were controlled to less than 5%. The dried OBPs were packed in aluminum zipper bags with desiccant and stored in a desiccator. Subsequently, the raw material formulations of the extrusion feed were mixed with brown rice in different ratios of OBP (5, 7.5, 10, 12.5, and 15%), respectively. Detailed grouping and composition as shown in Fig. 1.

### Determination of radial expansion ratio (ER)

ER was determined following the method described by Cheng et al. (2024a, b). Each group of random sampling (*n* = 20). Afterward, the diameter of the samples was measured using an electronic digital vernier caliper. The expanded diameter of the sample (mm) was determined by rotating the sample 90° and measuring it again, then averaging the results. Finally, the ER (mm/mm) was calculated by dividing the average expansion diameter of each group by the hole diameter (3 mm) of the extruder.

### Textural profiling analysis (TPA)

The TPA of the sample was determined using the methodology described by Huang et al. (2023) and Lee et al. (2024), with some modifications. This study used a texture analyser (TA-XT2, Stable Micro Systems, Godalming, UK) with a P/50 probe (*n* = 20), and the specific parameters included compression mode; pre-test speed was 1 mm/sec; test speed was 2 mm/sec; post-test speed was 10 mm/sec; distance was 2 mm; and trigger force was 5 g. The hardness (N) and fracturability values were determined for the samples. The unit of fracturability was defined as the number of positive peaks within the sample.

### Determination of bulk density (BD)

The sample’s BD was determined using the method described by Lin et al. (2023a, b, c) with minor modifications. Glass beads of known volume (290 mL) and a measuring cylinder (500 mL) were prepared, followed by sampling (5 g of each group) and weighing. The glass beads were initially placed at the bottom of the cylinder. Next, one layer of glass beads and one layer of samples were stacked on each other. The highest part of the cylinder was covered in glass beads, and the space inside was filled with glass beads. The measurement of the height of the cylinder was recorded, resulting in the calculation of BD by dividing the weight by the volume.

### Determination of β-glucan

The β-glucan in the samples was determined using the Megazyme β-glucan (Mixed Linkage) assay kit (Neogen Co. Lansing, MI, USA). The sample (100 mg) was weighed into a tube and extracted with 10 mL of 50% ethanol at 80 °C for 10 min, then centrifugation (1,000 ×*g* for 10 min) with a centrifuge (Megafuge 16R, Thermo Fisher Scientific, Waltham, MA, USA) to remove the supernatant, repeating this procedure twice. Then, the residue was added with 50% ethanol 0.2 mL mixed by oscillation, and then 20 mM phosphate buffer solution 4 mL was added and remixed. Next, the solution was heated in a boiling water bath for 1 min, then taken out, oscillated evenly, then placed in a boiling water bath for 2 min, and oscillated evenly again. Equilibrate the tubes in a 50 °C water bath, then add 0.2 mL of lichenase solution with shaking to mix, followed by reaction for 60 min in a 50 °C water bath. During the reaction, oscillation was required every 15 min for the enzymatic reaction to be fully activated. Afterward, 5 mL of 20 mM sodium acetate buffer solution was added to each tube and shaken evenly, allowing it cooled to 25 °C, then centrifuged (1,000 ×*g* for 10 min). Each sample was divided into 3 tubes (all containing 0.1 mL of the above supernatant), where 2 tubes continued to react with 0.1 mL β-glucosidase, while the remaining tube was added 0.1 mL of 50 mM acetic acid buffer solution as a blank value. Next, all tubes were added with 3 mL of glucose oxidase-peroxidase mixed buffer solution (glucose oxidase > 12,000 Unit (U)/L, peroxidase > 650 U/L, 4-aminoantipyrine (81.3 mg/L)), followed by a 50 °C water bath reaction for 20 min. Finally, the absorbance of each sample at 510 nm was determined using a microplate spectrophotometer (BioTek Epoch 2, Agilent Technologies, Inc., Santa Clara, CA, USA). The following equations were used to calculate β-glucan content in the samples.

1 $$ \begin{aligned} & \beta - glucan\:content(\% ,wet\:basis) \\ & \quad = E \times \:F \times \:94 \times \:\frac{1}{{1000}} \times \:\frac{{100}}{W} \times \:\frac{{162}}{{180}} \\ \end{aligned} $$

2 $$\beta\:-glucan\:content(\%,dry\:basis)=\frac{Equations\left(1\right)}{100-Moisture\:content\left(\%\right)}$$

where,

△E: absorbance value of the reaction solution -absorbance value of the blank solution F: Conversion factor for glucose content (µg) (as 100 µg glucose/100 µg glucose absorbance).

94: Volume correction factor (0.1 mL of analytical solution from 9.4 mL).

1/1000: Conversion value of µg and mg.

W: weight of test sample (mg).

162/180: glucose conversion factor.

### Determination of resistant starch (RS) and non-RS

The RS and non-RS determination was performed using the Megazyme^®^ RS assay kit (Neogen Co. Lansing, MI, USA) and following the standard operating procedures provided by the manufacturer. The sample (100 mg) was milled to 40 mesh, placed in a 50 mL centrifuge tube, and added 4 mL of pancreatic α-amylase reagent (10 mg/mL AMG). The mixture was mixed and reacted for 16 h at 37 °C in a water bath. 4 mL of ethanol (99%) was added and centrifuged (6,000 ×*g*, 10 min), then the supernatant was collected. The residue was mixed with 8 mL of 50% ethanol, centrifuged at 6,000 ×*g* for 10 min, and then separated and collected from the supernatant. This step was repeated twice. The above residue was mixed with 2 mL of 2 M potassium hydroxide, stirred in an ice bath for 20 min, and then added into 8 mL of acetic acid buffer solution (1.2 M, pH 3.8). Subsequently, 0.1 mL of the supernatant was incorporated with 3 mL GOPOD reagent, then reacted at 50℃ for 20 min while the absorbance was measured using a microplate spectrophotometer (BioTek Epoch 2, Agilent Technologies, Inc., Santa Clara, CA, USA) at 510 nm. As mentioned above, the standard was 0.1 mL of 100 mg/mL glucose, and the procedures were repeated. Subsequently, the RS content of the sample was ascertained by utilizing the subsequent formula:

3 $$ \begin{aligned} & \mathrm{Re} sis\tan t\:starch\;\left( {RS < 10\% ;\frac{g}{{100g\;sample}}} \right) \\ & \quad = Absorbance\:value\:of\:the\,(Sample - Control) \\ & \quad \times \frac{{Absorbance\:value\:of\;100\mu g\;glu\cos e}}{{100mg}} \times 9.27 \\ \end{aligned} $$

Regarding the determination of non-RS content, the above-prepared supernatant was repeated in the same manner as RS’s, and the following equation calculated the non-RS content in the sample.

### Determination of digestibility (In vitro)

The digestibility of the sample, which consisted of both rapidly digested starch (RDS), slowly digestible starch (SDS), and estimated glycemic index (eGI), was assessed using the methodology described by Wang et al. (2023). The mixture, consisting of 1.82 g of porcine pancreatic α-amylase reagent, was then supplemented with 6 mL of distilled water, subjected to stirring for 15 min, and centrifuged at 1,500 ×*g* for 10 min. Subsequently, 5 mL of supernatant and 62.5 µL of amyloglucosidase solution (AMG; 3,300 U/mL) were mixed uniformly for the preparation. Next, 100 mg of the sample was mixed with pH 5.2, 4 mL of 0.1 M sodium acetate buffer solution, and 1 mL of the amylase mentioned above. The mixture was placed in a water bath for 0, 20, 30, 60, 90, 120, 150, and 180 min (with 120 rpm shaking), followed by a sampling of 0.1 mL for each. Afterward, 0.9 mL of 95% ethanol was added to terminate the reaction. Then, centrifuged at 1,500 ×*g* for 10 min, 0.1 mL of the supernatant was taken, and 3 mL of glucose oxidase-peroxidase aminoantipyrine (GOPOD) reagent was added. The absorbance value was determined using a microplate spectrophotometer (BioTek Epoch 2, Agilent Technologies, Inc., Santa Clara, CA, USA) at a wavelength of 510 nm. The RDS, SDS and eGI of the sample were calculated using the following equations:

4 $$Rapidly\:digestible\:starch\left(RDS;\%\right)=\left(G20\right.-\left.G0\right)\times\:0.9$$

5 $$Slowly\:digestible\:starch(SDS;\%)=\left(G120\right.-\left.G20\right)\times\:0.9$$

6 $$ \begin{aligned} & Hydrolysis\:index\left( {HI;\% } \right) \\ & \quad = Area\:below\:hydrolysis\:curve\:of\frac{{Sample}}{{Standard}} \times 100 \\ \end{aligned} $$

7 $$ Estimate\:of\:the\:glycemic\:index\left( {eGI} \right) = 39.71 + 0.549 \times HI $$

### Sensory evaluation

The sensory evaluation methods were based on the descriptions of Huang et al. (2024a) with minor modifications. For this study, 30 panelists were randomly recruited based on consumer type (individuals who were not food science professional-trained personnel) and then evaluated the products based on preference and purchase intention. The evaluation was based on a 9-point Hedonic Scale, in which panelists evaluated the product’s appearance, aroma, off-flavor, fracturability, hardness, stickiness, flavor, and overall preferences. The score includes one point for immensely dislike, five for no comment (not about liking or disliking), and nine for extremely like. Before conducting sensory evaluations, we ensured that participants were given clear instructions regarding informed consent. We informed them of our adherence to established ethical guidelines and obtained their consent to participate in the evaluation. This approach was adopted to ensure that participants were fully informed about the process and could make an informed decision about whether to participate in this sensory evaluation.

### Statistical analysis

Each trial underwent at least three repetitions, and the outcomes were presented as the mean ± standard deviation (SD). This study used the statistical software of XLSTAT (version 2019, Lumivero, Denver, CO, USA) with the one-way variance analysis (ANOVA). Duncan’s multiple-range test was used to compare the groups’ variability. It was determined that the difference was significant when *p* < 0.05 was observed.

## Results and discussion

### Effect of different ratios’ oat by-product ratios (OBP) on appearance and physicochemical properties of extruded snacks

This study showed that the appearance of the snacks in each group under the same extrusion processing conditions varied depending on the incorporation of different ratios of OBPs (Fig. 1). Specifically, the 100% brown rice group exhibited the most extended length and maximum cross-sectional area. In contrast, the snacks experienced a reduction in size and flattening of cross-sectional diameters as OBPs incorporation increased. It was hypothesized that the high concentration of dietary fiber in OBP contributed to the snacks’ unappealing appearance. Namely, the high hydrophilicity of the fiber content affects the physical characteristics of the extruded snacks; in particular, ER decreases and thus increases hardness (Kalahal et al. 2024; Nikinmaa et al. 2023a). In addition, the effect of thermal treatment on the extrusion process depends on the different levels of lipids and proteins present (Blandino et al. 2022). Decreased starch pasting is observed in the case of lipids, and less starch swelling is observed in the case of high-protein contents (Dalbhagat et al. 2019). Conversely, it has been reported that modifications in the ratio of these ingredients incorporated as described above can also facilitate the drawbacks (Boakye et al. 2023). Specifically, appropriate fiber incorporation can provide large air pockets and thin cell walls to improve the fracturability and hardness of extruded snacks, while appropriate protein incorporation can inhibit air bubble formation to reduce the fracturability of extruded snacks (Boakye et al. 2023; Korkerd et al. 2016). Another possible explanation for this deficiency might be that the screw speed and temperature are currently suitable for use in the control group, which would necessitate improvement in the future following the determination of a suitable OBP incorporation ratio. However, optimizing each recipe individually is uneconomical and difficult to achieve in practice due to the many analyses required (Nikinmaa et al. 2023a) and the probability of subtle changes that may be undetectable to humans. Thereby, the BD and hardness of the extruded snacks were decreased by modifying the parameters such as die pressure, screw speed, temperatures, specific mechanical energy, torque, feed moisture, and feed speed (Boakye et al. 2023; Dalbhagat et al. 2019; Ying et al. 2017; Zambrano et al. 2024). It is important to note that the unappealing visual presentation and texture of OBP extruded snacks can significantly reduce consumer willingness to purchase them (Kojić et al. 2022; Nikinmaa et al. 2023a). However, it has been suggested that incorporating 10–30% cereal fiber into extruded product recipes as substitutes for flour can provide appropriate hardness and consumer acceptance (Hsieh et al. 1989; Korkerd et al. 2016; Rzedzicki et al. 2000).

**Table 1:** Effect of different ratios of freeze-dried (FD) and complex drying (CD; autoclaving (121 °C, 15 min) combined with hot-air drying) oat by-product (OBP) on the radial expansion ratio (ER) and texture properties (hardness and fracturability) properties of extruded snacks

Groups	Brown rice (%)	OBP (%)	Radial expansion ratio (ER; mm/mm)	Hardness (*N*)	Fracturability
Control	100.00	0.00	3.25 ± 0.14^a^	82.52 ± 8.99^d^	28.60 ± 2.91^a^
FD1	95.00	5.00	3.08 ± 0.16^a^	103.67 ± 11.76^d^	25.40 ± 3.54^ab^
FD2	92.50	7.50	2.76 ± 0.18^b^	113.82 ± 18.53^d^	23.60 ± 3.60^ab^
FD3	90.00	10.00	2.08 ± 0.11^c^	142.78 ± 23.92^cd^	19.73 ± 2.60^bcd^
FD4	87.50	12.50	1.69 ± 0.11^d^	192.72 ± 35.51^bc^	15.67 ± 3.13^cde^
FD5	85.00	15.00	1.59 ± 0.16^d^	220.04 ± 48.13^b^	14.20 ± 3.90^de^
CD1	95.00	5.00	3.08 ± 0.12^a^	96.63 ± 11.00^d^	22.60 ± 1.83^b^
CD 2	92.50	7.50	2.73 ± 0.2^b^	121.51 ± 14.68^d^	20.13 ± 3.60^bc^
CD 3	90.00	10.00	2.01 ± 0.15^c^	221.58 ± 48.13^b^	15.20 ± 2.91^cde^
CD 4	87.50	12.50	1.69 ± 0.14^d^	325.96 ± 57.33^a^	11.93 ± 1.40^e^
CD 5	85.00	15.00	1.51 ± 0.13^d^	327.82 ± 47.14^a^	11.67 ± 3.20^e^

Each value was expressed as mean ± SD (*n* = 3)

Lowercase letters with different superscripts in the same column represent a significant difference, *p* < 0.05

### Radial expansion ratio (ER) and texture (hardness and fracturability)

This study showed that the ERs of snacks with different ratios of OBP extrusion ranged from 1.51 to 3.08 mm/mm (Table 1), where the maximum and minimum ERs were found in the FD-OBP 5% group (3.08 mm/mm) and the CD-OBP 15% group (1.51 mm/mm), respectively. This also implied a significant decrease (*p* < 0.05) in ER for increasing the ratio of OBP incorporation, but an insignificant difference was observed between the 12.5 and 15% groups. It was hypothesized that a possible reason for the lower limit of the ER was reached with the 12.5% OBP incorporation. Anton et al. (2009) reported that soybean flour’s higher seed coat fiber contents also influenced the extruded product’s decreased expansion ratio. The authors have indicated that this is attributed to the fibers causing cell wall rupture of the product, thereby preventing the bubbles from inflating to their maximum potential. Studies have indicated that incorporating high-protein and high-fiber elements into starch-based formulations with extrusion processes reduces the snack’s overall porosity and increases its structural density (Korkerd et al. 2016; Onwulata et al. 2001).

In terms of textural properties, this study found that the control group showed the lowest hardness and the highest fracturability (Table 1), while the CD5 group exhibited the highest hardness but the lowest fracturability. Specifically, the hardness of extruded snacks increased according to the ratio of OBP incorporation. In comparison, fracturability shows the opposite trend. It is worth mentioning that there was a significant difference in hardness (*p* < 0.05), whereas fracturability was not significant between groups with OBP incorporation over 7.5%. These phenomena can be attributed to the textural properties of the extruded snacks correlated with BD (Dalbhagat et al. 2019). Specifically, incorporating proteins or fibers in the formulation leads to the formation of compression pores, which leads to a tighter tissue structure and fracturability (Ayoub et al. 2013; Beck et al. 2018).

### Bulk density

**Fig. 2:**
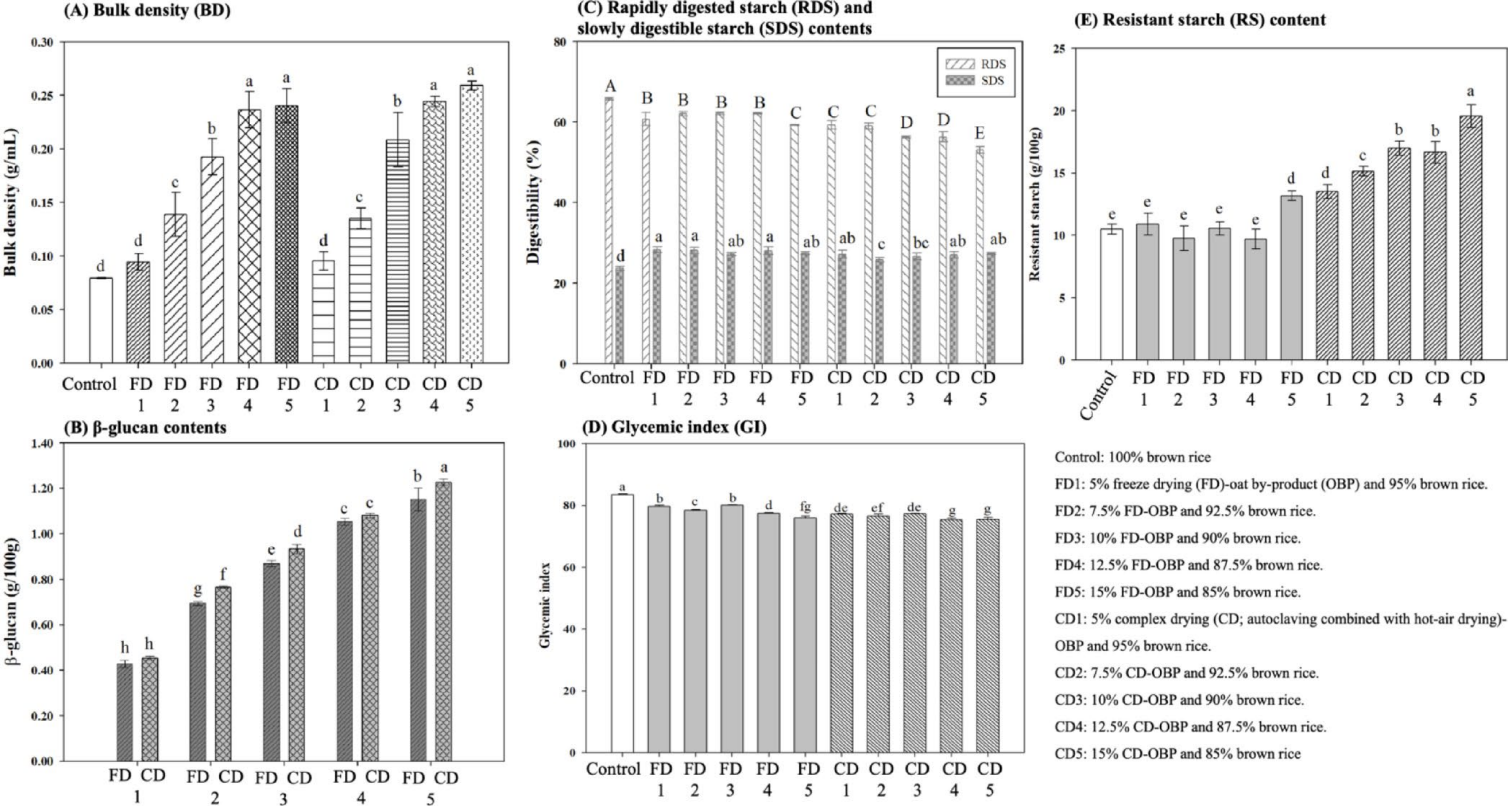
Effects of different ratios of freeze-dried (FD) and complex drying (CD; autoclaving combined with hot-air drying) of oat by-product (OBP) on the **A** bulk density (BD), **B** β-glucan content, **C** rapidly digested starch (RDS) and slowly digestible starch (SDS), **D** resistant starch (RS) content and (GI) value of extruded snacks.. Each value was expressed as mean ± SD (*n* = 3). Lowercase letters with different superscripts in the same column represent a significant difference, *p* < 0.05

BD is also one of the critical indices of consumer acceptability of extruded snacks (Jain et al. 2022). This study’s results indicated that the BD of each group was the minimum in the control group (0.079 g/mL) (Fig. 2A). In contrast, the remaining groups increased as the OBP incorporation ratio increased, whereas 15% of CD- and FD-OBPs groups exhibited the maximum BD but the minimum ER (Sect. 3.1.1 above). This can be attributed to the decreased density of extruded snacks associated with the increased ER, and this study’s findings were consistent with the trends reported by Pitts et al. (2014) and Sharif et al. (2014). In addition, BD was typically affected by being subjected to barrel temperature and screw speed precisely due to ER changes in cell structure, pores, and voids (Jain et al. 2022). Therefore, the above results showed that OBP incorporation ratio, BD, and hardness negatively correlated with ER and fracturability.

### β-glucan contents

This study showed that β-glucan content was positively correlated with the ratio of OBP incorporated (Fig. 2B). Specifically, the CD-OBP groups contained slightly more β-glucan than the FD groups, despite no statistically significant differences. In addition, Nikinmaa et al. (2023b) reported that the determined amount of β-glucan that can be extracted from the extrudes of whole-wheat oat-based matrices ranged from 0.60 to 1.31 g/100 g, suggesting that these results were similar to this study. Notably, the extraction rate of β-glucan was related to increased specific mechanical energy (SME) and β-glucan degradation during the extrusion process (Nikinmaa et al. 2023b).

### In vitro digestibility

In this study, the results showed that the incorporation of the CD-OBP groups resulted in significantly decreased RDS levels (*p* < 0.05) compared to the FD groups (Fig. 2C). It was hypothesized that the autoclaving treatment caused the amylose and amylopectin molecules to migrate, facilitating the amylose chain connections, destroying the original crystalline structure, and forming new ones (Dominguez-Ayala et al. 2023). Specifically, it has been reported that the enhanced mobility and interactions of molecular chains within the crystalline region encompassing starch granules have been found to play a role in stabilizing the hydrogen bonding between the starch chains (An et al. 2024; Dominguez-Ayala et al. 2023). In addition, compared to the control group, RDS decreased significantly, whereas SDS increased significantly (*p* < 0.05) for all groups, regardless of the type of OBP added (Fig. 2C). However, it has also been reported that the enhanced digestibility of extruded snacks was associated in some cases with starch digestibility due to starch melting and depolymerizing to a slight increase, thus leading to an adverse health risk in terms of glycaemic response (Roye et al. 2021). Moreover, the high temperature, high pressure, and shear force to which the starch is subjected during the extrusion process can damage the granular structure of the starch (Kumari et al. 2024). Additionally, these factors increase the degree of pasting, making it more susceptible to enzymatic hydrolysis while contributing to an increase in digestibility (Brennan et al. 2013; Soler et al. 2020). Another possible explanation may be that the proteins around starch particles in the mixture are exposed to high temperature and pressure, limiting starch granule swelling and starch pasting, starch structure formation as tight stacks (crystallinity) that are resistant to enzymatic hydrolysis (Huang et al. 2024c; Kaur et al. 2023). Remarkably, the tightly structured is appropriate for co-extruded snacks requiring filling (Blandino et al. 2022). Moreover, it has also been reported that dry heating, RDS in RS, creates more small molecular fractions with suitable chain lengths, enhancing better rearrangement within the starch molecules during annealing (Kumari and Sit 2023). In addition, freeze-drying can impact starch’s solubility, leaching, and damaged crystalline formation, leading to increased water absorption (Huang et al. 2024b; Soler et al. 2020).

Regarding the GI values, this study showed that the control group was significantly higher (*p* < 0.05) than the other groups by extrusion processing (Fig. 2D). Simultaneously, the GI values of the groups decreased as the OBP incorporated ratio increased. According to reports, elevating the ER of extruded snacks may increase starch hydrolysis, consequently leading to a swifter glucose response (Alam et al. 2016; Nikinmaa et al. 2023b). However, OBP is rich in dietary fiber (β-glucan), which might contribute to reduced starch digestibility by amylose-fat inclusion complexes externally wrapping the starch molecules and restricting access to hydrolytic enzymes (Qadir and Wani 2022). It also contributed to decreased RDS and GI, while SDS and RS remarkably increased and agreed with published previous results (Soler et al. 2020). Despite the current findings in this study indicate that the extruded snacks remain high-GI (≥ 70) foods (Capriles et al. 2021). However, the inclusion of OBP led to a reduction in GI compared to the control group. Consequently, the following action should explore the synergistic use of other methods, further modification of OBP, or adjustment of the extrusion process parameters to enhance RS levels.

**Fig. 3:**
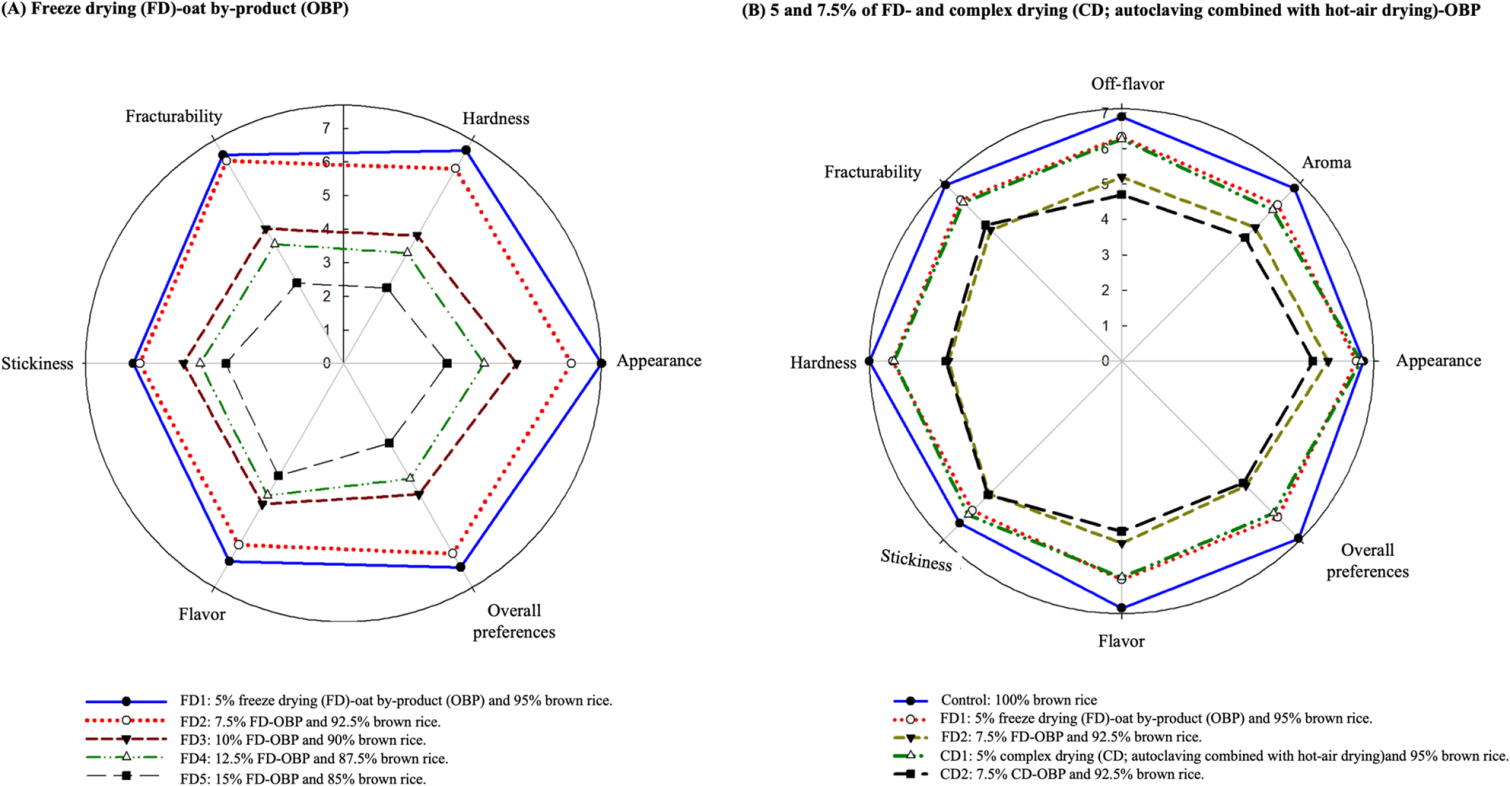
Sensory evaluations of (**A**) different ratios of freeze-dried (FD) of oat by-product (OBP) of extruded snacks and (**B**) 5 and 7.5% of FD- and complex drying (CD; autoclaving combined with hot-air drying)-OBP of extruded snacks

### Resistant starch (RS) content

This study showed that the RS content of the extruded snacks increased following the incorporation of the CD- and FD-OBP groups (Fig. 2E), particularly in the CD groups, with the increase in RS being significantly higher than in the FD groups (*p* < 0.05). More specifically, group CD5 exhibited the maximum RS content (19.55 g/100 g), while group F4 showed the minimum (9.69 g/100 g). This phenomenon can be explained by CD-OBP preparation belonging to the heat moisture treatment (HMT) class, namely, at low moisture (less than 35%) and 84–120 °C temperatures (Hoover 2010; Y.-W. Lin et al. 2023a, b, c; Soler et al. 2020). This results in changes in the amorphous and crystalline structure of the starch particles, thus increasing the RDS while modifying the physicochemical properties of the starch (Huang et al. 2016; Y.-W. Lin et al. 2023a, b, c; Soler et al. 2020). However, low-moisture (15 g/100 g) extrusion manufacturing has also been confirmed to increase the RS content in extruded snacks (Brahma et al. 2016; Nikinmaa et al. 2023b). Notably, drying at 90 °C and above has been reported to facilitate the formation of long-chain double-helix high-temperature-resistant ordered starch structures and be less favorable to forming amylose-lipid complexes in starch particles (Cheng et al. 2024a, b; Nikinmaa et al. 2023a; Thachil et al. 2014). This also implied that the starch showed an increased tolerance to digestive enzymes (Cheng et al. 2024a, b). Therefore, it was found that CD-OBP contributed positively to the increase of RS content caused by both high-temperature sterilization and hot air drying.

### Sensory evaluation

This study conducted the first stage of sensory evaluation for all the OBP groups with different ratios of extruded snacks by consumer-type sensory evaluation. It was confirmed that the overall preferences of the 5 and 7.5% groups incorporated in this study were significantly better than others (Fig. 3A). However, many panelists provided feedback indicating that the extruded snacks incorporating an OBP ratio over 7.5% were highly rigid in texture, difficult to chew, and suffered from issues such as stickiness and reversion flavor (from oxidized lipids). Therefore, in the second stage, FD- and CD-OBPs were selected as the 5 and 7.5% incorporation ratios compared with the control group for the preference evaluation. The effects of different drying treatments on the appearance, aroma, taste, fracturability, hardness, stickiness, flavor, and overall preferences of the OBPs on the extruded snacks were verified. This study showed that the appearance scores of the control, FD1, and CD1 groups were the same (Fig. 3B), but the scores were significantly different (*p* < 0.05) compared to FD2 and CD2 groups. Regarding aroma, the control group was the highest while the FD1 and CD1 groups were the second, and there was a significant difference between all groups (*p* < 0.05). Unfortunately, the same resulted in the off-flavor as well. It was hypothesized to be related to the property of brown rice to oxidize readily. This may be explained by the presence of lipids in the bran layer of brown rice, causing oxidization and negatively affecting sensory characteristics (Gondal et al. 2021; Y.-W. Lin et al. 2023a, b, c). However, it has been indicated that flavor issues stemming from substandard processing conditions can elevate the lipid oxidation rate of oats, resulting in undesirable attributes such as staleness and moldiness (Nikinmaa et al. 2023a; Zhang et al. 2024). In addition, scores in the fracturability, hardness, stickiness, flavor, and overall preferences groups followed the same trend as above. These results implicate that OBP incorporation over 5% would lead to substantially lower scores for all sensory parameters. Moreover, incorporating OBP prepared with different drying treatments revealed an insignificant difference in the scores of all sensory indicators for extruded snacks. This also implied that the impact on the extruded snacks was the ratio of the incorporated OBP. Therefore, the percentage of OBP that was the most preferred by the panelists in this study was 5%. Agnieszka et al. (2015) reported the best yield for incorporating 20% oat bran to extrude snacks, which performance received the approval of the trained panelists. Similarly, extruded snacks containing 5% citrus pomace fiber have been reported to have the highest level of acceptability in consumer evaluations (Zambrano et al. 2024), similar to this study’s results. Therefore, the panelists showed a preference for a 5% OBP.

### Estimated extruded snack production costs of oats by-products (OBP)

This study estimated the production cost of OBP extruded snacks, yet the investigation of this study involved the evaluation of the feasibility of production without entering into formal commercialization. Therefore, the subsequent costs of packaging, warehousing, shipment, sales, management, etc., in this study could not be estimated correctly, except for the preliminary calculation of the costs of raw materials and processes. This suggests that cost projections that have not been executed in practice are susceptible to estimation errors. The cost estimation findings from this study demonstrate that utilizing CD-OBP for production was the most economical option, as it yields the lowest production cost (Table 2). Therefore, this study verified that the repeated utilization of OBP confirms its ability to yield substantial advantages for the participating companies. Specifically, it enables the maximization of material utilization and contributes to offsetting (or amortization) the expenses associated with procuring oat raw materials.

**Table 2:** The estimated extruded snack production costs for 5% freeze-dried (FD)- oat by-product (OBP) and complex drying (CD; autoclaving combined with hot-air drying)-OBP

Extruded snack process cost	Item	USD $/kg
Material cost	Oats	1.37
	Oat by-product (OBP)	0.21
	Brown rice	3.12
		USD/kg/h
Power consumption	Freeze-drying (FD)	0.41
	Complex drying (CD; autoclaving combined with hot-air drying)	0.07
	Extrusion process	0.15
		USD/kg/h
Finished product cost	5% FD-OBP	15.08
	5% CD-OBP	14.99

Extruded snacks (Brown rice base) market price (USD $/kg): 86.00

Minus fixed costs such as packaging, management, shipment, warehousing, and sales (USD $/kg): 21.50

## Conclusions

The findings of this study suggest that integrating OBP into the circular bioeconomy for circular production within the industry presents a feasible solution. Given the paramount importance of ensuring the nutritional safety of food products, it is imperative to address the susceptibility of OBP’s high water content to quality degradation, thereby necessitating effective food waste management. In addition, this implies that without considering circular production, it would be necessary to dry the material for a certain period to prevent the proliferation of food-borne pathogens and then produce the extruded snacks under suitable conditions. Therefore, this study combined the formulation of soluble dietary fiber (β-glucan) enriched OBP with brown rice through extrusion processing to enhance the nutritional value of the extruded snack. This involved increasing RS content, reducing digestibility (in vitro), and lowering GI values. This study also determined that an optimal OBP incorporation of 5% and a discernible level of consumer willingness to purchase were identified through consumer-based sensory evaluations. However, to better understand the process and meet consumers’ practical demands (such as texture and mouthfeel), further investigation and improvement of the optimal formulation are essential. These implications also suggest a potential increase in consumer acceptance of this valuable food matrix and the facilitation of circular production on a commercial scale. However, further exploration of this concept holds promise for promoting sustainable production and enhancing the value of underutilized by-products. Therefore, this study’s findings will provide valuable insights into selecting extruded treatments for OBPs to enhance the quality of extruded snacks.

## Data Availability

Data will be made available on request.

## References

[CR1] Alam SA, Järvinen J, Kokkonen H, Jurvelin J, Poutanen K et al (2016) Factors affecting structural properties and in vitro starch digestibility of extruded starchy foams containing Bran. J Cereal Sci 71:190–197. 10.1016/j.jcs.2016.08.018

[CR2] An H, Ma Q, Zhang F, Zhai C, Sun J et al (2024) Insight into microstructure evolution during starch retrogradation by infrared and Raman spectroscopy combined with two-dimensional correlation spectroscopy analysis. Food Hydrocolloids 146:109174. 10.1016/j.foodhyd.2023.10

[CR3] Anton AA, Fulcher G, R., Arntfield SD (2009) Physical and nutritional impact of fortification of corn starch-based extruded snacks with common bean (Phaseolus vulgaris L.) flour: effects of bean addition and extrusion cooking. Food Chem 113(4):989–996. 10.1016/j.foodchem.2008.08.050

[CR4] Auestad N, Fulgoni VL (2015) What current literature tells Us about sustainable diets: emerging research linking dietary patterns, environmental sustainability, and economics. Adv Nutr 6(1):19–36. 10.3945/an.114.005694 25593141 10.3945/an.114.005694 PMC4288277

[CR5] Ayoub A, Liu Y, Miller DD, Rizvi SSH (2013) The effect of low shear on the development of fortified extruded rice products. Starch - Stärke 65(5–6):517–526. 10.1002/star.201200101

[CR6] Beck SM, Knoerzer K, Foerster M, Mayo S, Philipp C et al (2018) Low moisture extrusion of pea protein and pea fibre fortified rice starch blends. J Food Eng 231:61–71. 10.1016/j.jfoodeng.2018.03.004

[CR7] Blandino M, Bresciani A, Loscalzo M, Vanara F, Marti A (2022) Extruded snacks from pigmented rice: phenolic profile and physical properties. J Cereal Sci 103:103347. 10.1016/j.jcs.2021.103347

[CR8] Boakye PG, Okyere AY, Bharathi R, Murai T, Annor GA (2023) Physicochemical and nutritional properties of extruded products from cereals of the triticeae tribe – A review. Food Chem Adv 3:100379. 10.1016/j.focha.2023.100379

[CR9] Brahma S, Weier SA, Rose DJ (2016) Effects of selected extrusion parameters on physicochemical properties and in vitro starch digestibility and β-glucan extractability of whole grain Oats. J Cereal Sci 70:85–90. 10.1016/j.jcs.2016.05.001

[CR10] Brennan MA, Derbyshire E, Tiwari BK, Brennan CS (2013) Ready-to-eat snack products: the role of extrusion technology in developing consumer acceptable and nutritious snacks. Int J Food Sci Technol 48(5):893–902. 10.1111/ijfs.12055

[CR11] Capriles VD, Conti-Silva AC, Gomes Arêas JA (2021) Effects of oligofructose-enriched inulin addition before and after the extrusion process on the quality and postprandial glycemic response of corn-snacks. Food Biosci 43:101263. 10.1016/j.fbio.2021.101263

[CR13] Cheng Y-T, Huang P-H, Lu W-C, Chu S-C, Wang P-M et al (2023) Physicochemical properties of rainbow trout (Oncorhynchus mykiss) Filet treated with high-voltage electrostatic field under different storage temperatures. Front Sustain Food Syst 7:1158953. 10.3389/fsufs.2023.1158953

[CR12] Cheng G, Gu Z, Yang Y, Wang X, Zhao R et al (2024a) Understanding resistant-starch formation during drying high-amylose maize kernels. Int J Biol Macromol 260:129419. 10.1016/j.ijbiomac.2024.129419 38219936 10.1016/j.ijbiomac.2024.129419

[CR14] Cheng Y-T, Lu W-C, Chan Y-J, Huang P-H, Chiang P-Y et al (2024b) Effect of extruded Djulis (Chenopodium formosanum) snacks on the ameliorative potential against diabetic cardiomyopathy. J Funct Foods 116:106154. 10.1016/j.jff.2024.106154

[CR15] CNS (2019) CNS 2424 Brown rice http://www.cns-standards.org/CNS_standard.asp?CODE=CNS%202424 (accessed on 12 May 2024)

[CR16] Cooney R, de Sousa DB, Fernández-Ríos A, Mellett S, Rowan N et al (2023) A circular economy framework for seafood waste valorisation to Meet challenges and opportunities for intensive production and sustainability. J Clean Prod 392:136283. 10.1016/j.jclepro.2023.136283

[CR17] Dalbhagat CG, Mahato DK, Mishra HN (2019) Effect of extrusion processing on physicochemical, functional and nutritional characteristics of rice and rice-based products: A review. Trends Food Sci Technol 85:226–240. 10.1016/j.tifs.2019.01.001

[CR18] Do Q, Mishra N, Colicchia C, Creazza A, Ramudhin A (2022) An extended institutional theory perspective on the adoption of circular economy practices: insights from the seafood industry. Int J Prod Econ 247:108400. 10.1016/j.ijpe.2021.108400

[CR19] Dominguez-Ayala JE, Ayala-Ayala MT, Velazquez G, Espinosa-Arbelaez DG, Mendez-Montealvo G (2023) Crystal structure changes of native and retrograded starches modified by high hydrostatic pressure: physical dual modification. Food Hydrocolloids 140:108630. 10.1016/j.foodhyd.2023.108630

[CR20] Ek P, Baner JM, Ganjyal GM (2020) Chapter 8 - Extrusion processing of cereal grains, tubers, and seeds. In G. M. Ganjyal (Ed.), Extrusion Cooking (pp. 225–263). Woodhead Publishing. 10.1016/B978-0-12-815360-4.00008-0

[CR21] Gondal TA, Keast RSJ, Shellie RA, Jadhav SR, Gamlath S et al (2021) Consumer acceptance of brown and white rice varieties. Foods 10(8):1950. 10.3390/foods10081950 34441728 10.3390/foods10081950 PMC8391279

[CR22] Grasso S (2020) Extruded snacks from industrial by-products: A review. Trends Food Sci Technol 99:284–294. 10.1016/j.tifs.2020.03.012

[CR23] Hoover R (2010) The impact of heat-moisture treatment on molecular structures and properties of starches isolated from different botanical sources. Crit Rev Food Sci Nutr 50(9):835–847. 10.1080/10408390903001735 20924866 10.1080/10408390903001735

[CR24] Hsieh F, Mulvaney SJ, Huff HE, Lue S, Brent J (1989) Effect of dietary fiber and screw speed on some extrusion processing and product variables. LWT Food Sci Technol 22(4):204–207. 10.1016/0964-1955(92)90012-P

[CR29] Huang T-T, Zhou D-N, Jin Z-Y, Xu X-M, Chen H-Q (2016) Effect of repeated heat-moisture treatments on digestibility, physicochemical and structural properties of sweet potato starch. Food Hydrocolloids 54:202–210. 10.1016/j.foodhyd.2015.10.002

[CR28] Huang P-H, Hou C-Y, Hsieh C-W, Cheng K-C, Ciou J-Y et al (2023) Investigation of the physicochemical properties of the thin slices of dried pork meat paper mixed with squid. J Food Sci Technol 60(5):1590–1599. 10.1007/s13197-023-05702-6 37033313 10.1007/s13197-023-05702-6 PMC10076472

[CR25] Huang P-H, Cheng Y-T, Chan Y-J, Lu W-C, Li P-H (2024a) Effect of cooking treatment on the formation mechanism and physicochemical properties of mung bean (Vigna radiata L.) paste. J Agric Food Res 16:101054. 10.1016/j.jafr.2024.101054

[CR26] Huang P-H, Cheng Y-T, Chen S-J, Lu W-C, Chiang P-Y et al (2024b) Physicochemical properties of Dioscorea Alata Tainung 1 and 2 via different drying methods and application on the frozen Tangyuan. Food Bioscience 59:103993. 10.1016/j.fbio.2024

[CR27] Huang P-H, Chiu C-S, Chan Y-J, Su W-C, Wang C-CR et al (2024c) Effect of osmotic pressure and simultaneous heat-moisture phosphorylation treatments on the physicochemical properties of mung bean, water caltrop, and corn starches. Int J Biol Macromol 272:132358 10.1016/j.ijbiomac.2024.132358 38750862

[CR30] Jain R, Goomer S, Singh SN (2022) Mung-Oat snack of high protein content by twin screw extrusion using response surface methodology. Appl Food Res 2(1):100099. 10.1016/j.afres.2022.100099

[CR31] Jokinen I, Pihlava J-M, Puganen A, Sontag-Strohm T, Linderborg KM et al (2021) Predicting the properties of industrially produced oat flours by the characteristics of native oat grains or non-heat-treated groats. Foods 10(7):1552. 10.3390/foods10071552 34359422 10.3390/foods10071552 PMC8306198

[CR32] Jokinen I, Silventoinen-Veijalainen P, Lille M, Nordlund E, Holopainen-Mantila U (2023) Variability of carbohydrate composition and pasting properties of oat flakes and oat flours produced by industrial oat milling process—Comparison to non-heat-treated oat flours. Food Chem 405:134902. 10.1016/j.foodchem.2022.134902

[CR33] Kalahal S, Gavahian M, Lin J (2024) Development of innovative tigernut-based nutritional snack by extrusion process: effects of die temperature, screw speed, and formulation on physicochemical characteristics. Qual Assur Saf Crops Foods 16:1–22. 10.15586/qas.v16i1.1310

[CR34] Kaur P, Kaur H, Aggarwal R, Bains K, Mahal AK et al (2023) Effect of cooking and storage temperature on resistant starch in commonly consumed Indian wheat products and its effect upon blood glucose level. Front Nutr 10:1284487. 10.3389/fnut.2023.1284487 10.3389/fnut.2023.1284487 PMC10713747 38089929

[CR35] Kojić J, Belović M, Krulj J, Pezo L, Teslić N et al (2022) Textural, color and sensory features of spelt wholegrain snack enriched with betaine. Foods 11(3):475. 10.3390/foods11030475 35159625 10.3390/foods11030475 PMC8834531

[CR36] Korkerd S, Wanlapa S, Puttanlek C, Uttapap D, Rungsardthong V (2016) Expansion and functional properties of extruded snacks enriched with nutrition sources from food processing by-products. J Food Sci Technol 53(1):561–570 26787975 10.1007/s13197-015-2039-1 PMC4711464

[CR37] Kruijssen F, Tedesco I, Ward A, Pincus L, Love D et al (2020) Loss and waste in fish value chains: A review of the evidence from low and middle-income countries. Global Food Secur 26:100434. 10.1016/j.gfs.2020.100434

[CR38] Kumari B, Sit N (2023) Comprehensive review on single and dual modification of starch: Methods, properties and applications. Int J Biol Macromol 253:126952. 10.1016/j.ijbiomac.2023.126952 37722643 10.1016/j.ijbiomac.2023.126952

[CR39] Kumari S, Kaur BP, Thiruvalluvan M (2024) Ultrasound modified millet starch: changes in functional, pasting, thermal, structural, in vitro digestibility properties, and potential food applications. Food Hydrocolloids 153:110008. 10.1016/j.foodhyd.2024.110008

[CR40] Lee Y-C, Yu M-C, Yen C-Y, Tsay J-S, Hou C-Y et al (2024) Exploitation of post-ripening treatment for improving cold tolerance and storage period of Jin Huang Mango. Horticulturae 10(1):103. 10.3390/horticulturae10010103

[CR41] Lin C-H, Huang Y-T, Ciou J-Y, Cheng C-M, Wang G-T et al (2023a) Circular economy and sustainable recovery of Taiwanese tilapia (Oreochromis mossambicus) byproduct—The large-scale production of umami-rich seasoning material application. Foods 12(9):1921 37174458 10.3390/foods12091921 PMC10177915

[CR42] Lin Y-W, Tsai C-L, Chen C-J, Li P-L, Huang P-H (2023b) Insights into the effects of multiple frequency ultrasound combined with acid treatments on the physicochemical and thermal properties of brown rice postcooking. LWT 188:115423. 10.1016/j.lwt

[CR43] Lin YD, Huang PH, Chen YW, Hsieh CW, Tain YL et al (2023c) Sources, degradation, ingestion and effects of microplastics on humans: A review. Toxics 11(9):747. 10.3390/toxics11090747 10.3390/toxics11090747 PMC10534390 37755757

[CR44] Lucarini M, Zuorro A, Di Lena G, Lavecchia R, Durazzo A et al (2020) Sustainable management of secondary Raw materials from the marine food-chain: A case-study perspective. Sustainability 12(21):8997. 10.3390/su12218997

[CR46] Ma B-L, Zheng Z, Ren C (2021) Chapter 6 - Oat. In V. O. Sadras & D. F. Calderini (Eds.), Crop physiology case histories for major crops (pp. 222–248). Academic Press. 10.1016/B978-0-12-819194-1.000

[CR47] Mahadevan M, Calderini DF, Zwer PK, Sadras VO (2016) The critical period for yield determination in oat (Avena sativa L). Field Crops Res 199:109–116. 10.1016/j.fcr.2016.09.021

[CR49] Mansilla-Obando K, Llanos G, Gómez-Sotta E, Buchuk P, Ortiz F et al (2024) Eco-Innovation in the food industry: exploring consumer motivations in an emerging market. Foods 13(1):4. 10.3390/foods13010004 10.3390/foods13010004 PMC10778022 38201032

[CR50] McCarron R, Methven L, Grahl S, Elliott R, Lignou S (2024) Oat-based milk alternatives: the influence of physical and chemical properties on the sensory profile. Front Nutr 11:1345371. 10.3389/fnut.2024.1345371 10.3389/fnut.2024.1345371 PMC10877596 38379545

[CR51] Mohammed I, Forsido SF, Kuyu CG (2024) Optimization of barrel temperature and feed moisture content for better physicochemical and sensory properties of extruded snacks from blends of finger millet, sweet potato, and soybean composite flour using response surface methodology. Discover Appl Sci 6(4):183. 10.1007/s42452-024-05822-4

[CR52] Nikinmaa M, Mustonen SA, Huitula L, Laaksonen O, Linderborg KM et al (2023a) Wholegrain oat quality indicators for production of extruded snacks. LWT 174:114457. 10.1016/j.lwt.2023.114457

[CR53] Nikinmaa M, Zehnder-Wyss O, Nyström L, Sozer N (2023b) Effect of extrusion processing parameters on structure, texture and dietary fibre composition of directly expanded wholegrain oat-based matrices. LWT 184:114972. 10.1016/j.lwt.2023.114972

[CR54] Onwulata CI, Konstance RP, Smith PW, Holsinger VH (2001) Co-extrusion of dietary fiber and milk proteins in expanded corn products. LWT - Food Sci Technol 34(7):424–429. 10.1006/fstl.2000.0742

[CR55] Pitts KF, Favaro J, Austin P, Day L (2014) Co-effect of salt and sugar on extrusion processing, rheology, structure and fracture mechanical properties of wheat–corn blend. J Food Eng 127:58–66. 10.1016/j.jfoodeng.2013.11.026

[CR56] Qadir N, Wani IA (2022) In-vitro digestibility of rice starch and factors regulating its digestion process: A review. Carbohydr Polym 291:119600. 10.1016/j.carbpol.2022.119600 35698347 10.1016/j.carbpol.2022.119600

[CR57] Roye C, Van Wayenbergh E, Henrion M, De Bondt Y, Chanvrier H et al (2021) Extrusion-cooking affects oat Bran physicochemical and nutrition-related properties and increases its β-glucan extractability. J Cereal Sci 102:103360. 10.1016/j.jcs.2021.103360

[CR58] Rzedzicki Z, Szpryngiel B, Sobota A (2000) Estimation of some chosen physical properties of extrudates obtained from corn semolina and OAT Bran mixtures. Int Agrophys 14

[CR59] Sethi S, Tyagi SK, Anurag RK (2016) Plant-based milk alternatives an emerging segment of functional beverages: A review. J Food Sci Technol 53(9):3408–3423. 10.1007/s13197-016-2328-3 27777447 10.1007/s13197-016-2328-3 PMC5069255

[CR45] Shahbandeh M (2024) Oats production worldwide from 2015/2016 to 2023/2024 (in million metric tons) https://www.statista.com/statistics/1073536/production-of-oats-worldwide/#:~:text=In%202022%2F2023%2C%20the%20global%20production%20volume%20of,oats%20amounted%20to%20approximately%2025.13%20million%20metric%20tons. (accessed on 13 May 2024)

[CR60] Sharif MK, Rizvi SSH, Paraman I (2014) Characterization of supercritical fluid extrusion processed rice–soy crisps fortified with micronutrients and soy protein. LWT - Food Sci Technol 56(2):414–420. 10.1016/j.lwt.2013.10.04

[CR61] Sharma N, Yeasmen N, Orsat V (2024) Green extraction of Chickpea (Cicer arietinum)-based functional beverage: assessment of nutritional quality and storage stability. Food Bioscience 59:104237. 10.1016/j.fbio.2024.104237

[CR62] Silventoinen-Veijalainen P, Sneck A-M, Nordlund E, Rosa-Sibakov N (2024) Influence of oat flour characteristics on the physicochemical properties of oat-based milk substitutes. Food Hydrocolloids 147:109402. 10.1016/j.foodhyd.2023.10

[CR63] Soler A, Velazquez G, Velazquez-Castillo R, Morales-Sanchez E, Osorio-Diaz P et al (2020) Retrogradation of autoclaved corn starches: effect of water content on the resistant starch formation and structure. Carbohydr Res 497:108137. 10.1016/j.carres.2020.108137 32889435 10.1016/j.carres.2020.108137

[CR64] Sumargo F, Gulati P, Weier SA, Clarke J, Rose DJ (2016) Effects of processing moisture on the physical properties and in vitro digestibility of starch and protein in extruded brown rice and Pinto bean composite flours. Food Chem 211:726–733. 10.1016/j.foodchem.2016.05.097 27283689 10.1016/j.foodchem.2016.05.097

[CR65] Thachil MT, Chouksey MK, Gudipati V (2014) Amylose-lipid complex formation during extrusion cooking: effect of added lipid type and amylose level on corn-based puffed snacks. Int J Food Sci Technol 49(2):309–316. 10.1111/ijfs.12333

[CR66] Wang Y-C, Liang Y-C, Huang F-L, Chang W-C (2023) Effect of freeze–thaw cycles on physicochemical and functional properties of ginger starch. Processes 11(6):1828. 10.3390/pr11061828

[CR67] Wu J, Zhu K, Zhang S, Shi M, Liao L (2024) Impact of oat supplementation on the structure, digestibility, and sensory properties of extruded instant rice. Foods 13(2):217. 10.3390/foods13020217 38254518 10.3390/foods13020217 PMC10815101

[CR68] Yang Z, Xie C, Bao Y, Liu F, Wang H et al (2023) Oat: current state and challenges in plant-based food applications. Trends Food Sci Technol 134:56–71

[CR69] Ying D, Hlaing MM, Lerisson J, Pitts K, Cheng L et al (2017) Physical properties and FTIR analysis of rice-oat flour and maize-oat flour based extruded food products containing Olive pomace. Food Res Int 100:665–673. 10.1016/j.foodres.2017.07.062 28873735 10.1016/j.foodres.2017.07.062

[CR70] Zambrano Y, Mariotti-Celis MS, Bouchon P (2024) 3G extruded snacks enriched with Catechin for high antioxidant capacity. LWT 192:115674

[CR71] Zhang Y, Zhang M, Huo R, Bai X, Wang P et al (2024) Effects of stir-frying on the processing characteristics and in vitro digestion of oat flour during storage. LWT 199:116006. 10.1016/j.lwt.2024.116006

